# Costs of sialendoscopy and impact on health-related quality of life

**DOI:** 10.1007/s00405-018-5196-9

**Published:** 2018-11-14

**Authors:** Johanna Jokela, Riitta Saarinen, Antti Mäkitie, Harri Sintonen, Risto Roine

**Affiliations:** 1 Department of Otorhinolaryngology – Head and Neck Surgery, University of Helsinki and Helsinki University Hospital, Helsinki, Finland; 2 Division of Ear, Nose and Throat Diseases, Department of Clinical Sciences, Intervention and Technology, Karolinska Institute and Karolinska Hospital, Stockholm, Sweden; 3 Department of Public Health, University of Helsinki, Helsinki, Finland; 4 University of Helsinki and Helsinki University Hospital, Helsinki, Finland; 5 University of Eastern Finland, Kuopio, Finland

**Keywords:** Sialendoscopy, Sialadenitis, Quality of life, Health-related quality of life, Costs, Cost-effectiveness

## Abstract

**Purpose:**

To analyse costs related to the diagnosis and treatment of patients with sialolithiasis and sialadenitis managed with sialendoscopy, and to prospectively evaluate the impact of sialendoscopy on health-related quality of life (HRQoL) in a longitudinal follow-up study.

**Methods:**

All patients undergoing sialendoscopy or sialendoscopy-assisted surgery at a tertiary care university hospital between January 2014 and May 2016 were identified from a surgical database, and the direct hospital costs were retrospectively evaluated from 1 year before to 1 year after the sialendoscopy. The 15D HRQoL questionnaire and a questionnaire exploring the use of health care services during the preceding 3 months were mailed to the patients before sialendoscopy as well as at 3 and 12 months after the operation.

**Results:**

A total of 260 patients were identified. Mean total hospital costs, costs related to the sialendoscopy, and complications were significantly higher in sialolithiasis patients than in patients with other diagnoses. 74 patients returned the baseline 15D questionnaire, and 51 patients all three 15D questionnaires. At baseline, the dimensions “discomfort and symptoms” and “distress” were lower in patients than in age- and gender-standardised general population, but the total 15D score did not differ significantly. The dimension “discomfort and symptoms” improved significantly at 3 and 12 months postoperatively, and the mean total HRQoL score improved in patients with sialolithiasis at 3 months postoperatively.

**Conclusions:**

The costs related to sialendoscopy are substantial and the cost-effectiveness of sialendoscopy warrants further studies. However, sialendoscopy seems to reduce patients’ discomfort and ailments and to improve HRQoL at least in patients with sialolithiasis.

## Introduction

The development of sialendoscopy has offered a minimally invasive alternative to diagnose and treat obstructive sialadenitis. This has led to gland-preserving salivary gland surgery and reduced the need for salivary gland excisions [1]. Although designed for the treatment of sialoliths, sialendoscopy is currently used also to manage non-stone-related obstructive sialadenitis and inflammatory conditions [2–6].

The most common symptom of obstructive sialadenitis is recurrent, usually painful swelling of the salivary gland during meals, while chronic inflammatory disorders such as Sjögren’s syndrome, chronic recurrent parotitis, and juvenile parotitis are more often characterized by intermittent, mildly tender swelling of the gland(s) that sometimes persists for days [7, 8]. Both disorders may be complicated by bacterial infections [8]. While the therapeutic benefits of sialendoscopy have been demonstrated in many studies and reviews [9–11], the effects of sialadenitis and sialendoscopic intervention on patients’ health-related quality of life (HRQoL) have been investigated less often. Also, the costs related to the treatment of these patients and to sialendoscopy have not been comprehensively explored.

The effectiveness of treatment can be measured by evaluating the frequency and severity of the disease and by recording the pre- and postoperative symptoms. In addition, assessment of the HRQoL has gained growing interest during the preceding decade. According to the World Health Organization, the instrument assessing HRQoL must contain at least physical, mental, and social aspects [12]. Because there is no gold standard for assessing otorhinolaryngological patients’ HRQoL, it remains a challenge to choose a proper questionnaire to reflect patients’ QoL.

We designed an observational study to evaluate the health care costs and direct hospital costs as well as HRQoL of patients treated with sialendoscopy. For the assessment of HRQoL, we used the generic, self-administered 15D questionnaire, which has been shown to be sensitive in detecting changes in many patient groups and allows comparison of treatment effectiveness across different disorders [13].

## Materials and methods

Between January 2014 and May 2016, altogether 260 patients underwent sialendoscopy or sialendoscopy-assisted operation at the Department of Otorhinolaryngology—Head and Neck Surgery, Helsinki University Hospital (HUH), Helsinki, Finland. The indications for the intervention were recurrent swelling or pain of a major salivary gland(s). All patients were treated endoscopically or with endoscopy-assisted combined techniques with direct transoral or transfacial sialolith removal usually under local anaesthesia as a day surgery. We did not use balloon dilators, lasers, or intraductal stonebreakers.

In all, 188 out of 260 patients were preoperatively invited by mail to participate in a follow-up questionnaire study related to HRQoL and use of health care services. Patients under 15 years of age (*n* = 9) and patients with insufficient Finnish or Swedish language skills (*n* = 20) were excluded. Also, some patients were not reached preoperatively mainly because of changed operation schedules and, in few cases, because of missing contact information (*n* = 43). Follow-up questionnaires were sent at 3 and 12 months after the operation to those patients who had returned the first questionnaire. If the follow-up questionnaires were not returned, a reminder and a new questionnaire were sent once. Four patients who underwent subsequent sialadenectomy were excluded from the follow-up.

The study protocol was approved by the Research Ethics Committee of Helsinki University Hospital.

### Costs

Direct hospital costs were evaluated for all 260 patients for the period from 1 year before to 1 year after the sialendoscopy. The costs were obtained from the clinical patient administration system (Ecomed, Datawell Ltd., Finland), which routinely stores all costs of hospital care for each patient treated at Helsinki University Hospital. We collected all costs for each patient related to the diagnostics and treatment of salivary gland disease, including outpatient and emergency department visits, phone calls, pathology examinations, imaging and laboratory services, operations, inpatient care, and any complications of sialendoscopy.

To assess the self-reported costs of health care services related to patients’ salivary gland disorders, we used a specifically designed questionnaire in which patients evaluated the number of doctor or nurse appointments, phone calls, and laboratory services conducted in public or private health care as well as inpatient treatments and the number of sick-leave days due to salivary gland disorder during the preceding 3 months. Use of services and duration of sick leave were converted to monetary units based on the most recent Finnish data on standard unit costs from 2011 [14].

### 15D

To assess HRQoL, we used the 15D, which is a 15-dimensional, generic, comprehensive, standardised, and self-administered HRQoL instrument that can be used both as a profile and as a single index score measure (http://www.15d-instrument.net/15d) [13]. The 15D consists of the following 15 dimensions: moving, seeing, hearing, breathing, sleeping, eating, speaking, excretion, usual activities, mental functioning, discomfort and symptoms, depression, distress, vitality, and sexual activity. For each of these dimensions, the respondent chooses from among five scores the one best describing his/her current state of health (best score = 1; worst score = 5). Valuation of the 15D is based on the application of the multiple-attribute utility theory, where a utility or preference weight is obtained from the general public through a three-stage valuation procedure and used to generate the utility score, i.e., the 15D score, which is a single index score. The maximum score is 1 (no problems on any dimension) and the minimum score 0 (equivalent to being dead). The minimal clinically important change or difference in the 15D has been estimated to be ± 0.015 [15]. The 15D has been shown to be highly reliable and responsive to change, comparing favourably with other similar instruments [13, 16–18]. Patients’ HRQoL was compared at baseline with that of the general population. The 15D data for the general Finnish population were obtained from the large Health 2011 Health Examination Survey [19]. For comparison with the patients of this study, participants from the Helsinki and Uusimaa stratum of the Health 2011 survey were selected who were in the age range of the patients. This subsample (*n* = 1339) was weighted to reflect the age and gender distribution of the patients.

### Statistical analysis

Data were analysed with SPSS version 22 (SPSS Inc., Chicago, IL, USA). Categorical variables are reported as frequencies and percentages, and continuous variables as means (standard deviation, SD). Differences between the groups regarding age, and operation time were tested with one-way Anova with Bonferroni correction and regarding gender, gland, and diagnosis with Pearson Chi-Squared test. The significance of the difference in the mean 15D score and costs between different groups was analysed with independent samples *t* test, and the significance between baseline and follow-up 15D scores and costs with paired samples *t* test. *P* values < 0.05 were considered significant.

## Results

Hospital costs were calculated for the whole patient series (*n* = 260), which had a mean age of 46 years (SD 17.2, range 3.6 to 86.1). The postoperative diagnosis was chronic sialadenitis in 50%, sialolithiasis in 43%, stenosis/stricture of the main duct in 7.3%, and sialosis in 0.4% of patients. Thirteen patients underwent sialendoscopy twice, four patients underwent submandibulectomy, and two patients underwent sublingual gland excision during the study period. Patient demographics and characteristics are shown in Table 1.

**Table 1:** Patient demographics and characteristics

	All patients, *n* = 260	Patients with preoperative 15D data, *n* = 74	Patients with complete 15D data, *n* = 51	Patients with complete answers concerning the use of health care services, *n* = 43
*Sex, n* (%)				
Female	171 (66)	53 (72)	38 (75)	32 (74)
Male	89 (34)	21 (28)	13 (25)	11 (26)
Age, mean, years (SD)	46 (17.2)	51 (16.0)	53 (15.5)	53 (15.0)
*Gland, n* (%)				
Parotid gland	114 (44)	34 (46)	30 (59)	25 (58)
Submandibular gland	146 (56)	40 (54)	21 (41)	18 (42)
Bilateral sialendoscopy, *n* (%)	18 (6.9)	7 (9.5)	7 (14)	6 (14)
*Diagnosis, n* (%)				
Sialolithiasis	111 (43)	30 (41)	16 (31)	14 (33)
Sialadenitis	129 (50)	39 (53)	31 (61)	25 (58)
Duct stenosis/stricture	19 (7.3)	5 (6.8)	4 (7.8)	4 (9.3)
Sialosis	1 (0.4)	0 (0)	0 (0)	0 (0)
Operation time, mean, min (SD)	36 (27)	35 (26)	37 (29)	39 (31)

The 15D questionnaire and the questionnaire concerning self-reported use of health care services were sent to 188 patients. At baseline, 74 patients (39%), at 3 months 60 patients (32%), and at 12 months 56 patients (30%) returned the questionnaires. All three 15D questionnaires were returned by 51 patients (27%). Patient characteristics and demographics are shown in Table 1. The respondents and non-respondents did not differ significantly regarding mean age, gender, operated gland, postoperative diagnosis, or operation time.

### Costs

**Fig. 1:**
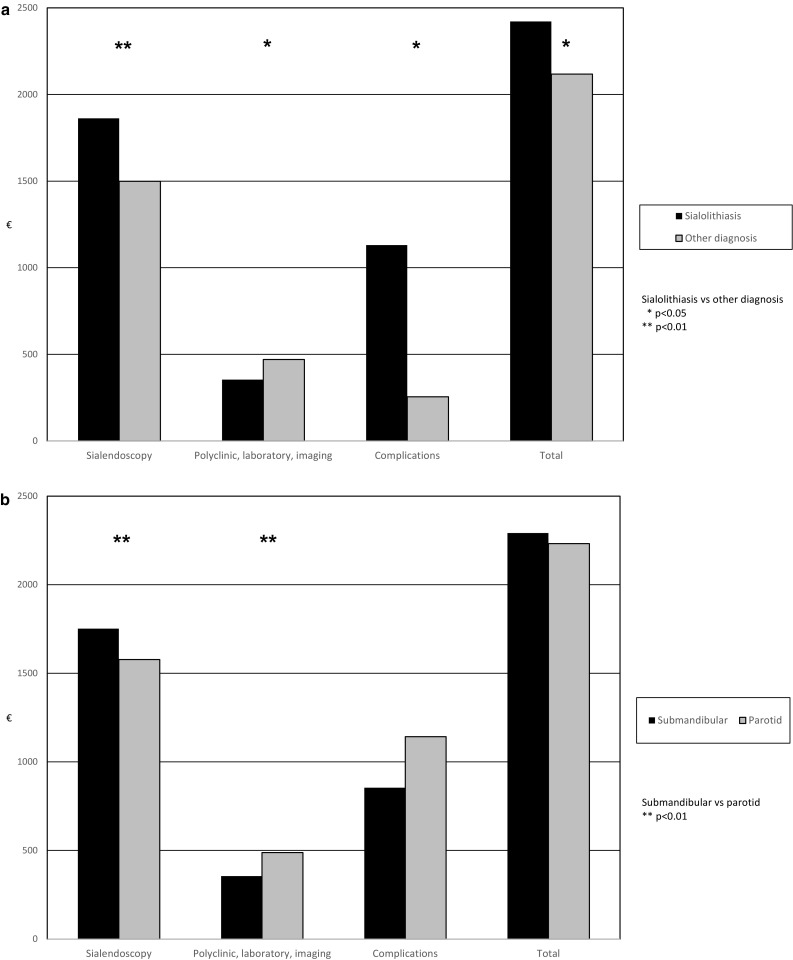
Differences in mean hospital costs during the two-year follow-up according to diagnosis (**a**) and treated salivary gland (**b**)

During the two-year time period, the total hospital costs were 2265 € per patient (SD 968 €, range 1280 to 7880 €), the costs of the sialendoscopic procedure(s) were 1673 € per patient (SD 466 €, range 1170–4373 €; 1593 € per procedure), and the costs of clinical encounters (including also costs related to phone contacts, laboratory, and imaging) were 416 € per patient (SD 378 €, range 0–2215 €). Both mean total hospital costs and mean costs of the operation were significantly higher in patients with sialoliths than in patients with other postoperative diagnoses (*p* = 0.014 and *p* < 0.001, respectively), while the mean costs of clinical encounters were significantly higher in patients without sialoliths (*p* = 0.012). The mean total hospital costs did not differ significantly between the patients with parotid problems and those with submandibular problems, but mean costs of the sialendoscopy were significantly higher in submandibular patients (*p* = 0.006) and mean costs of clinical encounters were higher in patients with parotid problems (*p* = 0.006). Patients’ age or gender did not affect the hospital costs. Complications causing direct hospital costs occurred in 21 cases. The mean cost per complication was significantly (*p* = 0.029) higher in patients with sialolithiasis (*n* = 16, 1131 €, SD 1444 €, range 110–4425 €) than in patients with other diagnoses (*n* = 5, 255 €, SD 130 €, range 110–435 €). The mean hospital costs are presented in Fig. 1.

**Fig. 2:**
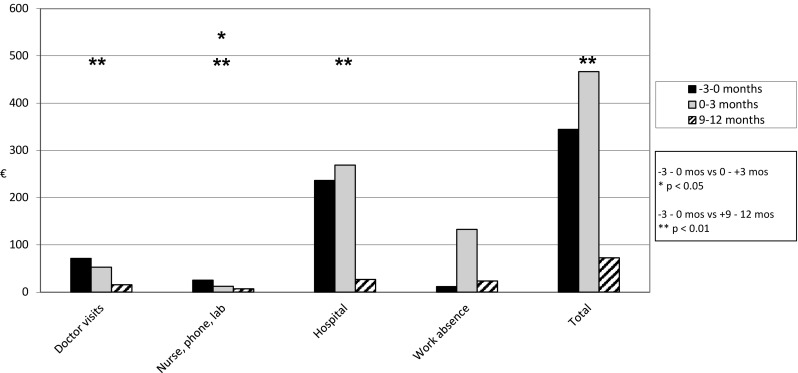
Mean costs of self-reported use of health care services during 3-month periods before and after sialendoscopy (*n* = 43)

Forty-three patients (23%) answered all three questionnaires concerning the use of primary health care services related to salivary gland disease during the preceding 3 months. The mean self-reported total costs of health care services and the productivity costs of sick leave were 345 € (SD 514 €) at baseline (during 3 months before sialendoscopy), 467 € (SD 1303 €) at 0–3 months postoperatively, and 72 € (SD 197 €) at 9–12 months postoperatively. The costs of the sialendoscopy are not included. A significant decrease in the mean total costs occurred from baseline to 12 months (*p* = 0.001). The costs of doctor visits in private or public primary health care, hospital costs, and costs of other contacts decreased significantly from baseline to 12 months postoperatively (*p* < 0.01) (Fig. 2).

### 15D

**Fig. 3:**
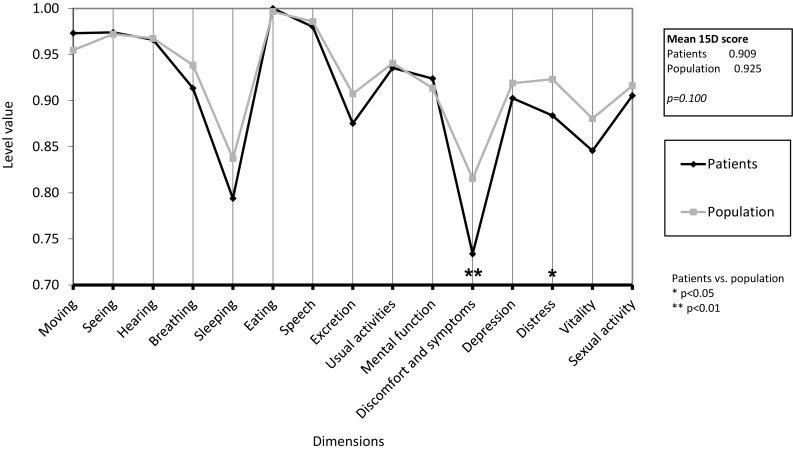
The mean 15D profile of patients (*n* = 74, mean age 51 years, 72% females) before sialendoscopy relative to that of the general population standardised for age and gender

At baseline, the mean total HRQoL score of the 74 patients (0.909, SD 0.079) was clinically importantly lower than that of the age- and gender-standardised general population (0.925, SD 0.025), but the difference was not statistically significant (*p* = 0.100). Of the individual 15D dimensions, the study cohort was significantly worse off than the general population on the dimensions of “discomfort and symptoms” (*p* = 0.002) and “distress” (*p* = 0.032) (Fig. 3).

**Fig. 4:**
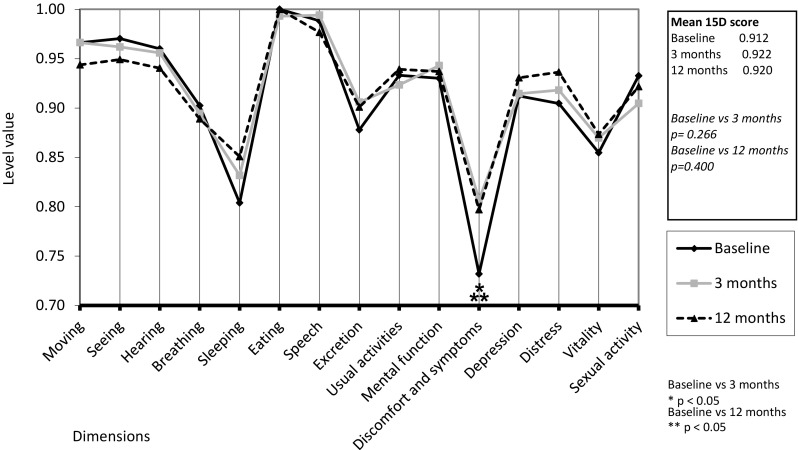
The mean 15D profiles of patients (*n* = 51, mean age 53 years, 75% females) before sialendoscopy and at 3 and 12 months postoperatively

Among the 51 patients who returned all three HRQoL questionnaires, a significant improvement was seen in the dimension of “discomfort and symptoms” at 3 months (*p* = 0.014) and at 12 months (*p* = 0.039) postoperatively, but not in the other dimensions or in the mean total HRQoL score (Fig. 4). The mean total HRQoL score did not differ significantly between parotid and submandibular gland patients or between diagnostic groups at any time point, but it was significantly improved in patients with sialolithiasis at 3 months postoperatively (*p* = 0.015). The mean scores of the dimension of “discomfort and symptoms” were significantly higher at 3 and 12 months postoperatively in submandibular gland patients (*p* = 0.022 and *p* = 0.021, respectively) than in parotid gland patients. The patients with sialolithiasis had significantly higher mean scores on the dimensions of “depression” and “distress” at baseline (*p* = 0.013 and *p* = 0.035, respectively) and on the dimensions of “discomfort and symptoms” (*p* = 0.046), “depression” (*p* = 0.002), and “distress” (*p* = 0.006) at 3 months postoperatively than patients with other diagnoses.

## Discussion

As new treatment methods are developed, it is important to evaluate their costs and value for HRQoL. This study investigated the costs of treatment of sialadenitis patients managed with sialendoscopy and measured the effect of sialendoscopy on patients’ HRQoL using the generic 15D questionnaire at a tertiary care academic hospital. This technique has been part of our management protocol for salivary gland disorders since 2005. Sialendoscopy comprised a substantial part of the total hospital costs of the treated patients. Both the mean total hospital costs and the mean sialendoscopy costs were higher in patients with sialolithiasis, while the mean costs of clinical encounters were higher in patients without sialoliths. Sialolithiasis patients more often needed general anaesthesia, overnight stay in hospital, or a second endoscopic procedure to ensure a successful stone removal, explaining the higher costs. Also, a higher rate of complications was encountered in these patients, which is in line with our earlier results [20]. The higher costs of clinical encounters in patients without sialoliths as well as in patients with parotid gland problems might be related to the over-representation of chronic sialadenitis in these patients with ongoing symptoms requiring ambulatory interventions such as intraductal saline or cortisone irrigations.

To our knowledge, there are no previous studies investigating the treatment costs in different diagnostic groups or between parotid and submandibular glands, and only few studies have evaluated the costs of sialendoscopy. Rosbe et al. [21] compared the average costs of care of patients with juvenile recurrent parotitis treated with sialendoscopy relative to those treated conservatively. The average costs in the sialendoscopy group were much higher ($31 338 per patient vs. $698 per patient), although the treatment outcomes did not differ significantly. However, the patients were not randomized and the sialendoscopy patients had a higher frequency and severity of symptoms, hindering comparison. Ong et al. [22] estimated the cost-effectiveness of transfacial sialendoscopy-assisted removal of parotid sialoliths by comparing it with the traditional parotidectomy performed for chronic parotitis. Complete or partial resolution of symptoms was noted in 87% of patients after transfacial resection and this procedure was less expensive ($22,482 vs. $30,546) and faster than parotidectomy. In our study, four patients underwent submandibulectomy with mean costs of 2293 € (not including the diagnostic costs and costs of clinical encounters) indicating that sialendoscopy (1673 €) is a more economical procedure in most cases if successful. In addition, according to current knowledge a minimally invasive treatment of the obstruction is recommended as recovery of secretory function is reached in most cases [23, 24].

Shashinder et al. [25] evaluated the average costs of transoral stone removal in their institution (£197) and deduced that this method is the most cost-effective relative to the sialendoscopy or gland resection. Their success rate for stone removal was 88% and improved over time. In our department, the average costs of transoral stone removal range from 330 to 1500 € depending on, e.g., the place of the procedure (outpatient department or operation theatre). A simple duct incision and stone removal are thus recommendable in ambulatory cases where the stone is palpable and distally located. Recently, Kowalczyk et al. [26] showed in a cost-effectiveness analysis based on a literature review that the upfront sialendoscopy is a more cost-effective treatment in radioiodine-induced sialadenitis than medical management utilizing ultrasound when the willingness-to-pay threshold is $50 000.

However, when assessing the cost-effectiveness of sialendoscopy or any other treatment, it is important to observe its possible effects on patients’ HRQoL. Patients’ and doctors’ perceptions of outcome might differ markedly, and the conception of HRQoL is highly individualistic. Moreover, sialendoscopy serves both as a diagnostic tool and a treatment method, usually simultaneously.

To date, no validated instrument exists for capturing salivary gland-specific HRQoL. Few studies have used generic instruments to assess the HRQoL of patients treated with sialedoscopy. Kroll et al. [27] evaluated patients’ HRQoL after sialendoscopy using the Short-Form-36 Health Survey (SF-36). In all, 80–85% of patients reported an improvement of symptoms, but still the patients showed worse values than the reference group in vitality and mental health. No pre-operative assessment was obtained. Two different studies have used the Glasgow Benefit Inventory (GBI) survey to assess possible improvement of patients` HRQoL after sialendoscopy. A positive effect was noted in both studies, comparing well with other otorhinolaryngological procedures where the GBI has been reported [28, 29]. Meier et al. [29] showed that the presence of stones as well as the examination of the parotid gland had a significant positive impact on GBI score.

The generic 15D instrument has been used to measure HRQoL among other benign otolaryngological conditions, such as juvenile recurrent respiratory papillomatosis, tonsillectomy, and septoplasty, as well as many other diseases [30–33]. In our study, the overall mean HRQoL score was clinically but not statistically significantly lower than in the age- and gender-standardised general population at baseline and had increased statistically significantly only in patients with sialolithiasis at the 3-month follow-up. However, sialendoscopy seems to reduce patients’ ailments since the score of the dimension “discomfort and symptoms” improved significantly after sialendoscopy in the whole patient population. The subgroup analysis revealed that ailments in sialolithiasis patients may be less burdensome and the sialendoscopy more effective than in patients with non-sialolith aetiology, while the patients with parotid problems might have more often ongoing symptoms after sialendoscopy compared to submandibular patients.

Efforts to construct a salivary gland-specific HRQoL instrument have been undertaken during the last years. In 2015, Gillespie et al. [34] used an adapted Oral Health Impact Profile-14 (OHIP-14) survey to measure patients’ salivary gland-related symptoms and HRQoL. Patients with sialoliths had significantly better salivary gland-related QoL after sialendoscopic-assisted operation than patients with non-sialolith aetiology, which is supported by our result. However, the retrospective, cross-sectional design of their study is a clear limitation. In 2016, Aubin-Pouliot et al. [35] introduced the Chronic Obstructive Sialadenitis Symptoms (COSS) questionnaire to quantify sialadenitis-specific symptoms and to assess the impact of sialendoscopy-assisted surgery. The COSS survey evaluates the sialadenitis symptoms and impact of the symptoms on daily functions and emotional well-being, measuring also the HRQoL [35, 36]. A prospective study consisting of 39 patients showed that patients with sialolithiasis as well as patients with submandibular problems showed a greater COSS score and symptom improvement than patients without sialolithiasis or patients with parotid symptoms, in accord with our findings [36].

The main limitation of our study was the low patient response rate to the questionnaires, which might be related to the study design, as patients received the questionnaires by mail and were requested to participate only once. This can lead to over- or underrepresentation of patients who were satisfied and experienced improvement of symptoms. Other methods such as electric questionnaires, an opportunity to participate in the study in the hospital on the operation day, and face to face contacting could have been more efficient. Also, the lack of a control group is a limitation. A control group could be, for instance, patients treated with transoral sialolith removal technique or a group treated conservatively. In addition, use of a disease-specific HRQoL measurement tool together with a generic one is recommended. Generic HRQoL instruments allow comparison to patients with other diseases or to the general population, while disease-specific instruments are more sensitive in detecting changes in symptoms as a consequence of treatment [37]. Validation of a universal salivary gland-specific HRQoL instrument is needed for better comparison between different techniques and studies.

## Conclusion

The sialendoscopic treatment of patients with sialolithiasis was more expensive than the treatment of patients with non-sialolith aetiology, while patients with parotid problems and patients with non-sialolith aetiology had higher costs of clinical encounters. The costs of sialendoscopy comprised a substantial portion of the total hospital costs of the treated patients during the two-year follow-up, highlighting the importance of patient selection. Sialadenitis patients experienced discomfort and distress more often than the age- and gender-standardised general population. Sialendoscopy reduced the ailments of the sialadenitis patients, but the overall HRQoL improved only in patients with sialolithiasis at the 3-month postoperative follow-up. Additional prospective, long-term follow-up trials are warranted to further assess the impact of sialendoscopy on the HRQoL as well as the cost-effectiveness of sialendoscopy.
